# Systematic review: the impact of exercise on mesenteric blood flow and its implication for preoperative rehabilitation

**DOI:** 10.1007/s10151-017-1589-9

**Published:** 2017-02-27

**Authors:** K. A. Knight, S. J. Moug, M. A. West

**Affiliations:** 1 Department of Surgery, Queen Elizabeth University Hospital, 1345 Govan Road, Glasgow, G51 4TF UK; 2 Department of Surgery, Royal Alexandra Hospital NHS Trust, Corsebar Road, Paisley, PA2 2PN UK; 3 Academic Unit of Cancer Sciences, Faculty of Medicine, University of Southampton, South Block, Mail Point 816, Southampton University Hospital, Southampton, SO16 6YD UK

**Keywords:** Colorectal, Cancer, Mesenteric blood flow, Prehabilitation

## Abstract

**Background:**

Exercise in the preoperative period, or prehabilitation, continues to evolve as an important tool in optimising patients awaiting major intra-abdominal surgery. It has been shown to reduce rates of post-operative morbidity and length of hospital stay. The mechanism by which this is achieved remains poorly understood. Adaptations in mesenteric flow in response to exercise may play a role in improving post-operative recovery by reducing rates of ileus and anastomotic leak.

**Aims:**

To systematically review the existing literature to clarify the impact of exercise on mesenteric arterial blood flow using Doppler ultrasound.

**Methods:**

PubMed, EMBASE and the Cochrane library were systematically searched to identify clinical trials using Doppler ultrasound to investigate the effect of exercise on flow through the superior mesenteric artery (SMA). Data were extracted including participant characteristics, frequency, intensity, timing and type of exercise and the effect on SMA flow. The quality of each study was assessed using the Downs and Black checklist.

**Results:**

Sixteen studies, comprising 305 participants in total, were included. Methodological quality was generally poor. Healthy volunteers were used in twelve studies. SMA flow was found to be reduced in response to exercise in twelve studies, increased in one and unchanged in two studies. Clinical heterogeneity precluded a meta-analysis.

**Conclusion:**

The weight of evidence suggests that superior mesenteric arterial flow is reduced immediately following exercise. Differences in frequency, intensity, timing and type of exercise make a consensus difficult. Further studies are warranted to provide a definitive understanding of the impact of exercise on mesenteric flow.

## Introduction

The systemic benefits of exercise have been recorded in the literature from as early as the time of Hippocrates [1]. Despite an acknowledgement throughout the centuries that exercise was necessary for the maintenance of health, it took until the twenty-first century for the idea that exercise can prevent disease to be formalised. In the 60 years that have elapsed since Morris and colleagues produced their landmark work “Coronary heart disease and physical activity of work” [2], which described lower rates of heart disease among physically active workers, strides have been undertaken in the use of exercise as preventative medicine. This is evidenced in the development of rehabilitation programmes following cardiac events which aim to reduce the likelihood of further events while returning patients to their baseline [3].

It has, however, taken somewhat longer for exercise to be recognised as a therapeutic tool which can be utilised as part of preoperative patient optimisation. General improvements arising from regular exercise including increases in muscle bulk, bone mineral density and strength are used in management of conditions such as osteoporosis [4, 5]. The cardiovascular adaptations resulting from regular aerobic exercise are also exploited as part of the spectrum of treatment options for hypertension, diabetes, cardio- and cerebrovascular disease and obesity [6–10].

More recently, use of exercise prior to an acute stressor such as surgery has emerged as a viable perioperative risk-reduction strategy [11, 12]. This concept, known as prehabilitation, was first used in sports medicine to reduce the impact of an injury prior to its occurrence. It has been explored as a method of preoperative optimisation in patients undergoing elective intra-abdominal surgery, most often major cancer resection [13–16]. These patients also frequently undergo pre- or post-operative chemoradiotherapy. Prehabilitation has been used to successfully mitigate the negative effects on physical fitness induced by such treatments [17, 18]. It is evolving to become a key part in the preoperative process for patients undergoing elective surgery [14, 16] and has been shown to aid return to baseline functioning [19], with further studies in progress examining its effect on post-operative outcomes.

The mechanistic link between physical exercise and improved outcome following colorectal surgery is yet to be fully elucidated. The changes occurring in the cardiorespiratory system in response to exercise have been studied in detail in a variety of training regimes [20–22]. The effect of exercise on mesenteric perfusion has been the subject of investigative research for over 60 years. However, the definitive impact of exercise, whether it be whole body involving large muscle groups or isometric involving specific muscles, on mesenteric blood flow continues to be a source of debate. Understanding the mechanisms by which preoperative exercise may improve outcome following colorectal surgery may in part lie in the response of the mesenteric vasculature. Bowel resection inevitably involves sacrificing a mesenteric vessel. If exercise improves mesenteric blood flow, it is conceivable that patients who exercise regularly may be less susceptible to complications following colorectal surgery such as delayed return of gut function, or even anastomotic breakdown.

The mesenteric circulation, comprising the superior and inferior mesenteric arteries arising directly from the abdominal aorta, is responsible for the delivery of arterial blood to the small bowel and colon. Multiple factors influence flow through these vessels, including central haemodynamics, autonomic stimulation and circulating hormones [23–25]. In athletes, the prevalence of lower gastrointestinal symptoms such as abdominal pain, diarrhoea and rectal bleeding suggests an alteration in flow during intense exercise [26, 27]. Other work has suggested that flow to the lower GI tract is preserved or even increased in the context of exercise [28–30].

The method of assessment of mesenteric haemodynamics has also varied over time, with invasive techniques involving cannulation of splanchnic vessels predominating in earlier years prior to the advent non-invasive imaging with Doppler ultrasonography. This improved the tolerability and feasibility of studies examining mesenteric flow. The aim of this systematic review was therefore to clarify the effects of exercise on the mesenteric circulation as assessed by Doppler ultrasound.

## Methods

A systematic search of EMBASE, PubMed and Cochrane databases was performed with the assistance of a medical librarian. Clinical trials involving both healthy volunteers and patients using Doppler ultrasound to investigate the effect of exercise on superior mesenteric arterial (SMA) perfusion were included. The inferior mesenteric artery was not chosen as the target vessel due to difficulties in accurately identifying this on non-invasive imaging such as Doppler. Review articles which did not present original data were excluded. The hypothesis was that exercise improved SMA blood flow in healthy volunteers and patients when measured by Doppler ultrasonography, compared with flow at rest. The primary outcome was change in SMA flow in response to exercise.

### Search strategy

PubMed (1950 to May 2015), EMBASE (1950 to May 2015) and Cochrane (1993 to May 2015) were searched using terms predefined by the reviewing authors (KK, MW). An update to the search using the same criteria was performed on 23 February 2016 in an attempt to capture any recently published literature. A hand search of the literature was conducted by the lead author using the reference lists of relevant original articles. Screening of each abstract was undertaken independently by two reviewers (KK, MW). Consensus was reached between reviewers on the suitability for inclusion. Full text versions of the included papers were then obtained and reviewed against the inclusion and exclusion criteria stated below. Both reviewers extracted the data from each included study using a predefined proforma.

### Inclusion criteria

Those studies examining the effect of exercise on mesenteric blood flow in adults aged 18 or over were eligible for inclusion. Only studies utilising Doppler ultrasound for measurement of mesenteric flow were included. The target vessel had to be the superior mesenteric artery (SMA), avoiding confusion between the portal and mesenteric systems.

### Exclusion criteria

Studies using animals were not included. Those not written or available in the English language were also excluded, as were papers arising from expert opinion. The search was limited to papers published after 1945, when Doppler ultrasound was introduced to clinical practice.

### Data extraction and analysis

The characteristics of each study, including the journal and country of publication, the study design and outcome measure, were extracted. The participant characteristics collected were type (healthy volunteer or patient), age, gender, height and weight. The primary outcome variable was SMA flow. The exercise outcome data extracted were: type, duration and intensity of exercise, pretest conditions, timing of measurements of SMA flow, flow parameter used and the effect on SMA perfusion. Meta-analyses were planned if sufficient clinical and statistical homogeneity was identified.

### Quality assessment

The Downs and Black checklist [31] was used to assess the quality of each study. This tool scores articles over 5 domains (reporting, bias, confounding factors, external validity and power) to produce a numerical value out of a possible 30 points. The studies were scored independently by two authors (KK, MW), and discrepancies were resolved by discussion.

## Results

### Data presentation and analysis

“Appendix” details the search strategies across all three databases. The initial literature search produced 275 abstracts (225 PubMed, 49 EMBASE and 1 Cochrane). This is presented in Figure 1 as per the Preferred Reporting Items for Systematic Reviews and Meta-Analyses (PRISMA) guidelines. Ten duplicates were identified and subsequently removed. Two reviewers (KK, MW) independently screened all candidate abstracts. Eighteen full text reviews were undertaken, with two papers excluded at this stage, as they did not fulfil the inclusion criteria. Sixteen remained and were included in the review. It was not possible to perform a meta-analysis of the data due to statistical heterogeneity across the included studies.

### Included studies

Sixteen studies, comprising 305 participants in total, were reviewed. Four studies enrolled patients, while twelve studies examined healthy volunteers. No restriction was placed on type of disease or exercise. The terms mesenteric and splanchnic are often used interchangeably when referring to colonic blood supply and were therefore included in the search strategy. All studies were single centre. There were no randomised controlled trials. The median number of patients in each was 12.

### Quality assessment

The Downs and Black checklist [31] was used to assess the quality of each included study. The median score, reflecting methodological quality, was 12 points. The study containing the most participants (59 triathletes) scored the highest at 19 points [32], while the paper scoring the least at 10 points is that widely cited as a seminal work in the study of mesenteric haemodynamics during exercise [28]. All of the included studies scored poorly in relation to power and internal validity due to confounding factors.

### Participant data

One hundred and eighty-eight of 305 participants were male. It was not possible to determine the exact gender balance due to two studies in which gender was not stated, and a further study in which it was not clear due to exclusions. Twelve of sixteen studies recruited healthy volunteers. This ranged from untrained individuals to endurance athletes. One study examined patients with autonomic failure exclusively [33]. A further three studies compared patients with chronic disease to age-matched controls: two examining the effects of exercise in patients with primary autonomic failure, and one study on patients with chronic heart failure [34–36]. Age range across the studies was variable. Study participants were significantly older in the trials where patients were recruited with a range from 46 to 72 years, while those recruiting healthy volunteers ranged in age from 20 to 51 years. Study and patient characteristics are summarised in Table 1.

**Table 1:** Study and participant characteristics

References	Country	Journal	Patient number	Patient group	Mean age/weight/ height
Peripheral and central vascular conductance influence on post-exercise hypotension Endo et al. [30]	Japan	J Physiol Anthropol	8	Healthy volunteers (5 males: 3 females)	20–32 years 60 ± 3 kg 169 ± 4 cm
Relationship between reduced lower abdominal blood flows and heart rate in recovery following cycling exercise Osada et al. [37]	Japan	Acta Physiol	11	Healthy male volunteers	24.7 ± 4.8 years 69.2 ± 10.9 kg, 170.4 ± 8.6 cm
Are splanchnic hemodynamics related to the development of gastrointestinal symptoms in Ironman triathletes? Wright et al. [32]	South Africa	Clin J Sport Med	59	Endurance athletes	Symptomatic group: 37.3 ± 9.2 years Controls: 42.3 ± 8.8 years
Differential arterial blood flow response of splanchnic and renal organs during low-intensity cycling exercise in women Endo et al. [29]	Japan	Am J Physiol Heart Circ Physio	8	Female healthy volunteers	21–30 years 53 ± 4 kg 161 ± 4 cm
Reproducibility of ultrasound blood flow measurement of the superior mesenteric artery before and after exercise Peters et al. [38]	The Netherlands	Int J Sports Med	12	Male healthy volunteers	25.9 ± 3.8 years 73.2 ± 8.4 kg $184.3 \pm 4.2\;{\text{cm}}$
Involvement of the human splanchnic circulation in pressor response induced by handgrip contraction Waaler et al. [41]	Norway	Acta Physiol Scand	7	Healthy students (1 male: 6 females)	23 ± 1 years 63 ± 13 kg 170 ± 7 cm
Reduced blood flow in abdominal viscera measured by Doppler ultrasound during one-legged knee extension Osada et al. [43]	Japan	J Appl Physiol	18	Male healthy volunteers	29 years (20–38) 67 kg (59–73) 170 cm (159–178)
Mesenteric, coeliac and splanchnic blood flow in humans during exercise Perko et al. [39]	Denmark	J Physiol	19	Healthy volunteers	28 years (24–35) 81 kg (57–85) 182 cm (173–191)
Hypotensive and regional haemodynamic effects of exercise, fasted and after food, in human sympathetic denervation Puvi-Rajasingham et al. [33]	UK	Clin Sci (Lond)	12	Patients with autonomic failure (5 males: 7 females)	56 years 78 kg (52–82)
Abnormal regional blood flow responses during and after exercise in human sympathetic denervation Puvi-Rajasingham et al. [44]	UK	J Physiol	17	Eleven patients with autonomic failure Six age-matched controls	Patients: 57 ± 6 years Controls: 56 ± 9 years
Systemic and regional (including superior mesenteric) haemodynamic responses during supine exercise while fasted and fed in normal man Puvi-Rajasingham et al. [34]	UK	Clin Res	10	Healthy volunteers	32 years (22–60) 62 kg (50–77)
Influence of central command and ergoreceptors on the splanchnic circulation during isometric exercise Duprez et al. [42]	Belgium	Eur J Appl Physiol Occup Physiol	10	Healthy volunteers	21.1 years 68 kg 179 cm
Priority of blood flow to splanchnic organs in humans during pre- and post-meal exercise Eriksen and Waaler [28]	Norway	Acta Physiol Scand	5	Healthy volunteers	Median age 23
Abnormality of superior mesenteric artery blood flow responses in human Chaudhuri et al. [35]	UK	J Physiol	23	Thirteen patients autonomic failure; ten age-matched controls	Patients: 56 years (46–72) Controls: 52 years (36–68)
Regional blood flow in chronic heart failure: the reason for the lack of correlation between patients’ exercise tolerance and cardiac output? Muller et al. [36]	UK	Br Heart J	40	Thirty patients with chronic heart failure (27 males) Ten healthy volunteers (8 males)	Patients: 67 years Controls: 49 years
Effects of exercise on mesenteric blood flow in man Qamar and Read [40]	UK	Gut	46	Healthy volunteers 16 fasting exercise, 15 exercise + meal, 15 meal alone (28 males: 18 females)	Fasting group: 25.6 years (19–52) 65.9 kg (52–86) Meal group 25.5 years (19–36) Control group 26 years (21–39)

### Type of exercise

The characteristics of the exercise regimes employed in the studies varied widely in terms of frequency, intensity, timing and type. Table 2 describes the details of the exercise intervention used in each study. Cycling at a stated intensity (steady state exercise) for a predetermined duration was the exercise mode of choice for nine studies [28–30, 33, 34, 37–39, 44]. Treadmill exercise was used in two studies [36, 40]. Of the remaining studies, three examined mesenteric haemodynamics in response to isometric exercise [35, 41, 42], one study was based around a triathalon [32], and one examined the response to knee extension–flexion at a stated intensity [43].

**Table 2:** Exercise intervention details according to the frequency, intensity, timing and type (FITT) principle

References	Frequency	Intensity	Timing	Type
Endo et al. [30]	Single episode	60% of heart rate (HR) reserve	60 min	Ergometer cycling
Osada et al. [37]	3 × 12 min session at different target intensities	30, 50 and 85% VO_2_max	3 min each at 1/3 then 2/3 max intensity, 6 min at target intensity	Ergometer cycling
Wright et al. [32]	–	–	–	Triathlon: 3.8 km swim 180 km cycle 42.2 km run
Endo et al. [29]	×3 interspersed with 30 min rest periods	40 W	4 min	Ergometer cycling
Peters et al. [38]	x2 interspersed with 5 min rest period	70% VO_2_max	30 min	Ergometer cycling
Waaler et al. [41]	x2 pressor tests separated by 10 min interval Repeated after 30 min rest	40% max voluntary contraction	2 min	Sustained handgrip
Osada et al. [43]	Ten cycles per minute	Low-intensity exercise (HR <90 beats/min)	20 min	Knee extension–flexion
Perko et al. [39]	Two episodes	75% VO_2_ max	Not stated	Fasting and postprandial ergometer cycling
Puvi-Rajasingham et al. [33]	2 × 9 min session (fasting and postprandial)	25, 50 and 75 W (3 min each)	9 min	Supine cycling
Puvi-Rajasingham et al. [44]	Single session	25, 50 and 75 W (3 min each)	9 min	Supine cycling
Puvi-Rajasingham et al. [34]	Two sessions separated by 2 days	25, 50 and 75 W (3 min each)	9 min	Ergometer cycling
Duprez et al. [42]	Single episode	30% maximal voluntary contraction	90 s	Ischaemic handgrip
Eriksen and Waaler [28]	Two sessions separated by 8 min rest	50–65 W and 150–200 W	4 min each	Semi-supine cycling
Chaudhuri et al. [35]	Single session	1/3 maximal pressure	120 s	Isometric exercise
Muller et al. [36]	Single session	2.7 km/h at varying slope angles (0, 1.3, 2.7)	4 min at each angle	Submaximal treadmill exercise
Qamar and Read [40]	Single session	5 km/h 20% gradient	15 min	Walking (treadmill)

The conditions in which the exercise was undertaken were also diverse (Table 3). In two studies, no pretest details were divulged [32, 37], but in the remaining studies the participants were required to fast. The duration of fasting ranged from 3 h pretest to 12 h; seven studies stated that patients fasted overnight. A pretest supine rest was mandatory in seven studies with duration varying from 20 to 40 min across the studies. Two studies precluded exercise in the 24 h prior to testing, while the remaining seven studies did not state whether a pretest rest was undertaken.

**Table 3:** Results according to impact on mesenteric flow

References	Pretest conditions	Target vessel	Parameter studied	Effect on perfusion
*Increased SMA flow*				
Eriksen and Waaler [28]	Fasted for 12 h 20 min reclining rest pretest	SMA	Flow = product of average velocity and vascular cross-sectional area Vascular conductance = flow/MAP	↑SMA flow following fasting exercise, SMA conductance ↓ after exercise in fed state but flow maintained at resting values
*Unchanged SMA flow*				
Endo et al. [30]	3 h fast Supine 40 min	SMA (renal, brachial & femoral)	SMA blood flow (vascular conductance)	Vascular conductance of SMA same as pre-exercise levels
Endoet al. [29]	3 h fast 24 h no exercise	SMA, renal and splenic arteries	SMA RI (MBV/MAP)	SMA MBV close to resting values SMA RI unchanged
*Reduced SMA flow*				
Qamar and Read [40]	Overnight fast 30 min supine rest	SMA	No details	SMABF ↓ in fasting state, mild ↑following exercise + meal, SMABF ↑ at 5 min but no other time in meal only group
Puvi-Rajasingham et al. [44]	Overnight fast Omitted medication on day of test	SMA	Flow = *πr* ^2^ x TAV x 60 SMA vascular resistance = MAP/Flow	SMA blood flow fell in controls throughout exercise, while only reduced in patients with AF after 9 min
Chauduri et al. [35]	Overnight fast Fludrocortisone stopped 48 h prior	SMA	SMA flow = *πr* ^2^ x TAV x 60 ml/min	No significant change in SMA flow or resistance in patients with sympathetic failure; ↑SMA resistance and ↓flow in controls
Osada et al. [37]	No details	Abdominal aorta and femoral arteries	SMA flow (blood velocity, vessel diameter)	Reduced blood flow as VO_2_ max increased but ↑ blood flow below 30% VO_2_ max
Wright et al. [32]	No details	SMA and coeliac arteries	Vessel diameter, systolic and diastolic velocity, resistance index	↓ diameter and RI with ↑diastolic velocity post-race
Peters et al. [38]	Fasted 3 h No exercise 24 h	SMA	Blood flow rate = TAMV x π x 4^−1^ x d^2^	Blood flow decreased immediately after exercise by 49% and 38%
Waaler et al. [41]	Fasting 12 h 30 min rest	SMA	SMA conductance = SMA flow/MAP	Reduced SMA vascular conductance during pressor response; less marked postprandially
Osada et al. [43]	10 h fast pretest	Abdominal aorta and femoral arteries	Visceral blood flow = BF aorta–(BF_RCFA_ + BF_LCFA_)	Visceral blood flow dropped significantly even at low work rates
Perko et al. [39]	Overnight fast pretest 30 min supine rest	SMA	Blood flow = TAMV x π x 4^−1^ x d^2^	25% reduction in mesenteric flow during submaximal cycling
Puvi-Rajasingham et al. [33]	Medication omitted 72 h fasted 30 min supine rest	SMA	SMA vascular resistance = MAP/flow	Slower increase in SMA vascular resistance in AF following fasted exercise; Less SMA vasoconstriction during postprandial exercise
Puvi-Rajasingham et al. [34]	Test 1: overnight fast Test 2: 30 min following liquid meal (500 kCal)	SMA flow and vascular resistance	Flow = *πr* ^2^ x TAV x 60 SMA vascular resistance = MAP/Flow	↑SMA flow at rest, ↓SMA flow during exercise
Duprez et al. [42]	Overnight fast	SMA	Pulsatility index	↓SMA PI during and at end of exercise

### Effect of feeding

In six studies, exercise was undertaken initially in fasting subjects then repeated following the ingestion of food [28, 33, 34, 39–41]. Meals were adjusted for size and energy content (1100–1700 kCal) depending on the participant’s weight in two studies [28, 41], while a further three studies used commercially available liquid meals varying in energy content from 390–550 kCal [33, 34, 40]. Perko [39] gave a 1000 kCal meal as standard. All studies included a 30 min rest period prior to undertaking exercise after meal ingestion. Both Waaler [41] and Perko [39] found that the reduction in SMA flow was less marked when exercising in the fed state compared with fasting. However, a further two studies, both conducted by the same group, found the opposite: SMA flow was reduced in exercise undertaken in both the fasting and fed state [33, 34]. Eriksen [28] found a moderate increase in SMA flow during exercise, but flow was unchanged in the fed state. Finally, Qamar reported a fall in SMA blood flow during exercise in the fasting state but an increase with exercise following meal ingestion [40].

### Assessment of perfusion

Only studies using Doppler ultrasound to assess SMA haemodynamics were included. In two studies, the flow in the superior mesenteric artery was estimated by measuring blood flow in the aorta and both femoral arteries [37, 43]. Otherwise, the SMA was the target vessel. A variety of correlates of flow were used across the included articles: impedance, conductance, pulsatility index, velocity, vessel diameter and cross-sectional area. One study gave no details on the method of determination of SMA flow [40].

### Timing of assessment

Muller [36] did not comment on the timing of the measurements of mesenteric flow in relation to the exercise performed. Across the remaining studies, the point in time where mesenteric flow was measured varied widely, ranging from 3 min following cessation of exercise to multiple measurements made over time extending up to 45 min. The timing and frequency of assessments are outlined in Table 4.

**Table 4:** Timing of assessments of mesenteric flow

Study	Pre-exercise assessment	Assessment during exercise	Post-exercise assessment
Endo et al. [30]	25–40 min	–	Post 1: 15-30 min Post 2: 40-45 min
Osada et al. [37]	Time not stated	–	Every 45 s until 3 min, alternate minutes between 4–14 min
Wright et al. [32]	Up to 3 days prerace	–	Upon race completion
Endo et al. [29]	Not stated	Ten points in first 2 min; every 30 s for 2 min	Every 30 s for 3 min
Peters et al. [38]	Three times in 25 min rest period	–	Test 1: immediately following 30 min cycling Test 2: following 20 min cycling
Waaler et al. [41]	2 min intervals for 5 min	During last 20 s of 2 min test	2 min intervals for 5 min
Osada et al. [43]	One resting measurement	Every 5 min for 20 min	At 1 min, between 2 and 5 min
Perko et al. [39]	One resting measurement	One measurement during exercise	2 min after cycling
Puvi-Rajasingham et al. [44]	Time not specified	At 3, 6 and 9 min	At 2, 5 and 10 min
Puvi-Rajasingham et al. [33]	Time not specified	At 3, 6 and 9 min	At 2, 5 and 10 min
Puvi-Rajasingham et al. [34]	Time not specified	At 3, 6 and 9 min	At 2, 5 and 10 min
Duprez et al. [42]	Continuously 3 min	Continuously for 90 s	Continuously for 3 min
Eriksen et al. [28]	During final 2 min of 20 min rest	During the final 2 min at end of 4 min cycle	During final 2 min of 8 min rest period
Chauduri et al. [35]	10 min prior	At 120 s	–
Muller et al. [36]	–	–	–
Qamar et al. [40]	After 30 min rest	–	*T* = 0, 5, 10, 15, 30 min

*AF* autonomic failure, *BP* blood pressure, *CO* cardiac output, *MAP* mean arterial pressure, *MBV* mean blood volume, *LCFA* left common femoral artery, *RCFA* right common femoral artery, *RI* resistance index, *SMA* superior mesenteric artery, *SMABF* superior mesenteric artery blood flow, *SVR* systemic vascular resistance, *TAMV* time-averaged mean velocity, *TAV* time-averaged velocity, *USS* ultrasound

### Effect on mesenteric perfusion

Table 3 summarises the studies by impact on SMA flow. Muller [36] did not produce definitive data clarifying their findings of the effect of exercise on mesenteric perfusion. Twelve of the remaining fifteen studies found mesenteric perfusion to be lower during or following exercise [32–35, 37–44]. Higher levels than resting flow were noted in one study [28]. It was unchanged in two studies [29, 30].

## Discussion

This systematic review sought to clarify the effect of exercise on flow through the superior mesenteric artery. Based on the available evidence, a definitive statement of the true impact of acute exercise on the mesenteric vasculature remains difficult. Basic exercise physiology favours the position that aerobic exercise results in reduced flow through the mesenteric arterial system in order to meet the demands of exercising muscle during and immediately after exercise. Exercising patients preoperatively can improve their physiological performance [13]. By exposing the SMA-dependent colon to acute reductions in flow, regular exercise may also condition the colon to situations such as surgical resection where flow will be reduced. The longer-term adaptations at cellular level in this setting are unknown, but increased ability to extract oxygen from the circulating arterial blood could be key to optimising anastomotic healing in patients undergoing colonic surgery.

The majority of the literature reviewed supports the theory that SMA flow is reduced in response to acute exercise. However, it remains unclear whether this is true for all types of exercise. The included studies used different intensities, durations and types of exercise with measurements made at varying intervals. This makes comparison of outcomes challenging. Therefore, a general consensus on the effect of exercise on mesenteric flow must be interpreted carefully in the context of these variables.

### Effect of intensity on SMA flow

It has long been suggested that mesenteric blood flow is reduced in proportion to the intensity of exercise [45–47]. The prevalence of GI symptoms in endurance athletes supports this theory [48–50]. Intensity varied widely across the studies and was recorded in different ways. In studies of dynamic exercise, it was expressed as a function of VO_2_ max [37–39], energy conversion in watts [28, 29, 33, 34, 44] and as the speed and gradient of incline on walking [36, 40]. A further two studies related exercise intensity to heart rate, expressed as beats per minute [43] and percentage heart rate reserve [30]. In those involving isometric exercise [35, 41, 42], intensity was measured as a percentage of maximal voluntary contraction. These measurements are not directly interchangeable, and therefore, the relationship between intensity and SMA flow requires consideration of these variables.

The effect of exercise at different intensities on SMA flow was examined in five studies [28, 33, 34, 37, 44]. Puvi-Rajasingham [34] used the same protocol of graded exercise with increments of 25 watts in three studies, including healthy volunteers and patients with autonomic failure (AF) [33, 44]. It was not possible to take measurements during exercise in one study and post-exercise measurements taken at 2 min were used as a surrogate [33], producing results which could not be assessed in relation to increasing intensity. SMA blood flow was progressively reduced throughout the graded exercise protocol in controls in the study involving healthy volunteers [34] while only reduced at the highest intensity in patients with AF [44]. The lack of sympathetic stimulation in the latter group would explain this finding with slower change occurring due to cellular release of circulating chemical agents.

Osada [37] found a similar trend in mesenteric haemodynamics when measuring abdominal blood flow in response to 12 min of ergometer cycling in healthy volunteers. At 30% VO_2_max, blood flow was slightly increased, while at 50% VO_2_max it was reduced by one-third and at 85% VO_2_max by 89%. Blood flow in this study was calculated by subtracting flow in the proximal right femoral artery from that in the abdominal aorta superior to the coeliac axis. While this indirect measure of mesenteric flow produces an estimation of SMA flow in contrast with other more direct measures, the results are similar, demonstrating clearly the compensatory decrease in SMA flow to facilitate redistribution of cardiac output as intensity rose.

The increase, although small, in flow at low-intensity contrasts with the moderate increases in SMA flow recorded in Eriksen’s study of semi-supine cycling at 50–65 and 150–200 W [28]. Measurements of SMA flow were taken in both the fasting and fed state. While exercise induced an increase in splanchnic vascular resistance and thereby reduced vascular conductance, it was not sufficient to reduce SMA flow. This was true in the fasted and postprandial state. The authors attributed their novel findings to the direct method of SMA flow measurement, the brief duration of exercise (4 min) and the submaximal intensity of exercise. Exercise of similar intensities was utilised in several studies [37–39], all of which found SMA flow to be reduced. Duration of exercise was indeed more prolonged in these studies, ranging from 12 to 30 min. Two studies also used direct measurement of the SMA to provide flow rates [38, 39]. The reproducibility of Eriksen’s findings has not been established in a study using similar duration, intensity and type of exercise. It remains a finding of note but contrasts with the body of evidence supporting decreased flow during exercise [32–34, 37–43].

On balance, the evidence suggests that flow through the SMA is reduced as exercise intensity increases. The findings of Eriksen and Waaler suggest that this may not be uniformly applicable, but the mechanism of increased SMA flow in response to exercise remains a finding which requires further studies of similar methodology to validate.

### Type of exercise

Table 2 demonstrates the different types of exercise used. Twelve employed dynamic exercise [28–30, 32–34, 36–40, 44], three isometric exercise [35, 41, 42] and one resistance exercise [43]. Dynamic exercise has been discussed in detail above, and therefore, here isometric and resistance exercise is examined.

The effect of sympathoneural activation during isometric exercise on mesenteric flow was examined in three studies [35, 41, 42]. Isometric exercise produces a sudden and significant cardiovascular stress [51]. In contrast to dynamic exercise, isometric exercise co-activates vagal and sympathetic responses [37]. Mean arterial pressure, cardiac output and heart rate increase as isometric contractile force increases [52]. Weipert suggested that isometric contractions may trigger a stronger chemoreflex response due to metabolite accumulation which raises blood pressure via sympathetic vasoconstriction [53].

Chauduri [35], Waaler [41] and Duprez [42] reported a reduction in SMA blood flow in response to sustained submaximal handgrip. Intensity in Duprez’s [42] and Chauduri’s [35] experiments was set at 30% maximum voluntary contraction, while Waaler [41] used 40% MVC. At intensities between 40 and 60% MVC, blood flow within the isometrically-contracting muscle reduces significantly or ceases completely, resulting in greater accumulation of metabolites to activate chemoreceptors. Duration was shorter at 90 s in Duprez’s study but similar in the others at 120 s [35, 41]. Chauduri included patients with autonomic failure alongside age-matched controls [35]. SMA blood flow was unchanged in the patient group, most probably due to a lack of vasoconstrictor nerve activity, while SMA constriction occurred during isometric exercise in healthy volunteers; the constriction therefore was likely to have been mediated neurally. Duprez found that the pulsatility index of the SMA was reduced in response to isometric exercise, translating to splanchnic vasodilatation, while Waaler noted vascular conductance to fall secondary to SMA vasoconstriction. Overall, the effect of isometric exercise on mesenteric flow was mediated by sympathetic nerve activity and found to be uniformly reduced.

Osada [43] examined blood flow in response to knee extension–flexion exercise against loads corresponding to increasing intensities of 2.1, 5.4, 10.3 and 15.2 W over a 20 min period. Reduced visceral blood flow was recorded, despite exercise intensity not being sufficient to raise the heart rate above 90 beats per minute. At heart rates of less than 90 bpm, the role of circulating catecholamines in mediating vasoconstriction is likely minimal, as demonstrated by Breuer [54]. Resistance exercise causes pronounced increases in both systolic and diastolic blood pressure resulting from sympathetic vasoconstriction, mechanical compression of blood vessels within the exercising muscle and the Valsalva manoeuvre generated [55]. The metabolite-induced vasodilatation within muscle stimulates group IV afferents, while group III afferents are excited by the mechanical distortion [56]. Low levels of dynamic exercise have been shown to activate group III and IV afferent fibres [57]. This in turn maintains cardiac output at a level sufficient to maintain the elevated blood pressure required to perfuse the dynamically exercising muscle [58]. There is a paucity of data examining the mechanism between the chemo- and mechanorececeptor reflex and its impact on regional flow, particularly with regard to the mesenteric circulation. No other studies were identified which used resistance exercise while examining flow. However, low-intensity dynamic exercise in women was found to result in unchanged rather than reduced flow [29]. Whether the mechanism resulting in suppression of SMA flow in exercise utilising isolated muscle groups differs from that in more generalised dynamic exercise remains to be elucidated and indeed validated by other studies.

### Timing of measurements

It is known that adaptations in the splanchnic circulation are most pronounced in the early phase of exercise [59]. The precise timing of measurements of SMA flow therefore has significant bearing on the result given the minute-to-minute variation in central and splanchnic haemodynamics. It is worth noting, particularly with regard to those studies making limited measurements, that these were static measurements of dynamic processes. Thus, having multiple points of assessment throughout exercise and the period thereafter produces a trend that enables interpretation of the changes occurring throughout.

Measurements were taken at various intervals across the studies (Table 4). Both studies which found SMA flow to be unchanged following exercise contrasted in the time points chosen for assessment, despite originating from the same authors [29, 30]. Endo [30] recorded measurements between 15 and 30 min and again at approximately 40 min following 60 min of ergometer cycling. Subjects were resting in the supine position during this time. The delay between cessation of exercise and measurement of SMA flow means that haemodynamics in the immediate period following exercise are not captured. The resulting data are likely to reflect recovery of the splanchnic circulation following exercise rather than adaptations in the acute phase. Endo [29] also measured the resistance index of the SMA in response to 4 min of ergometer cycling in a separate study. Measurements were taken at 14 points during exercise and at 6 points in the first 3 min following exercise. The findings were similar: the resistance index of the SMA was essentially unchanged. These studies contrast both in their methodology and in the timing and calculation of SMA flow, with the former using indirect measures (Table 1). The consistency of the finding of unchanged flow does suggest that timing alone cannot explain this. The much shorter duration of exercise coupled with the low intensity in the latter study could contribute to this finding, while the former study’s late measurements could explain the perceived lack of change in mesenteric haemodynamics.

Isometric exercise lends itself more easily to frequent measurements due to its static nature. Duprez [42] obtained measurements continuously before, during and after exercise. Waaler [41] measured SMA conductance at 2 min intervals during the pre- and post-exercise period and obtained a single measurement during exercise. Chauduri [35] took measurements before and immediately upon cessation of 120 s of isometric exercise. Across these three studies, SMA flow was reduced in healthy volunteers, regardless of method of measurement. While the timing of measurement is only one variable influencing the interpretation of SMA flow data, the recording of flow correlates throughout the exercise period and thereafter produces a trend which supports the consensus that in isometric exercise, SMA flow falls.

Of sixteen studies, only one (Eriksen [28]) reported increased SMA flow during exercise. Flow was measured before, during and after exercise consisting of semi-supine cycling at 50–65 and 150–200 W for 4 min each. Interestingly, their finding of increased SMA flow following exercise in the fasting state is to our knowledge unique. This has not been previously recorded in fasting exercise, only in postprandial exercise [41]. This study was small, with only five participants. Measurements were taken in the final 2 min of the resting, exercise and recovery phases (Table 4). SMA conductance fell during exercise in the postprandial state, but flow rates were maintained at pre-exercise levels despite this. The authors suggested the short duration of exercise and submaximal intensity may account for their findings. Other studies of similar exercise duration included Osada [37], Endo [29] and Muller [36] (Table 3). Intensity varied across these three studies. Endo [29] reported unchanged flow and Osada [37] found flow to be reduced, while Muller produced data for SMA blood flow at rest only. The significant heterogeneity in methodology and results renders interpretation of the physiological mechanism underlying Eriksen’s finding of increased flow almost impossible. Further studies would be required of similar intensity, duration and type of exercise to validate this.

### Special circumstances: fasting and postprandial exercise

Meal ingestion is known to be a potent splanchnic arterial vasodilator in humans [60] and results in a notable reduction in the superior mesenteric artery pulsatility index [61]. Six studies examined splanchnic haemodynamics in response to exercise in the fasting and fed state [28, 33, 34, 39–41]. Meal composition was tailored to individual size and weight in two studies [28, 41], while the others used commercially available liquid meals. Energy content varied from 390 to 1700 kCal across the studies. Qamar [40] gave a liquid meal during the first 10 min of exercise. Otherwise, meals were given at 30 min pre-exercise. Aside from Eriksen’s study [28], all uniformly found SMA flow to be reduced by exercise. Qamar [40] reported a temporary and modest increase in flow at 5 min following postprandial exercise. Flow was found to be more profoundly suppressed in fasting exercise, with less of a reduction noted postprandially [33, 34, 39, 41]. It can be presumed on the basis of these data that exercise in the fed state confers the benefit of postprandial vasodilation that protects against the increased mesenteric vascular tone induced by exercise. Digestion-induced vasodilatation is likely to result from the combined effects of local hormones [39], sympathetic stimulation [34] and meal load. When combined with exercise, these studies support the theory that exercise has little or no effect on SMA resistance in the postprandial state.

### Strengths and weaknesses of the systematic review

This article provides a contemporary review of the literature to date examining the effect of exercise on flow through the superior mesenteric artery. It was conducted in a systematic and rigorous fashion to ensure all relevant articles were identified. Using two investigators to screen each abstract and eligible full text paper against predefined criteria minimised bias. Using the SMA as the target vessel ensured distinction from the portal venous system which has also been studied in relation to exercise but does not solely give information on the blood supply to the bowel.

There were, however, limitations. All studies included were small (*n* < 60) and single centre, bringing into question both internal and external validity. Methodological quality was also poor overall. Twelve of sixteen studies were also conducted more than 15 years ago, suggesting waning interest in this important area. Well-designed, randomised controlled trials examining both the effect of acute exercise and regular exercise training on the mesenteric circulation are required to provide an up-to-date consensus, which will go some way to helping us understand and exploit the use of exercise in the preoperative period.

## Conclusion

The literature on the impact of exercise on mesenteric flow in man spans more than four decades and encompasses various frequencies, intensities, timings and types. A variety of different methods of assessment have been and continue to be used, with Doppler ultrasound remaining a reliable and convenient method of non-invasive assessment. It is at times difficult to perform accurate Doppler studies during exercise due to breathing artefact, particularly at higher workloads. More recently, magnetic resonance imaging of the splanchnic system is evolving to produce very detailed and accurate estimations of flow. Taking into account the heterogeneity observed across the studies in this review, a unifying statement is difficult. However, the majority of evidence supports the consensus that superior mesenteric arterial flow is reduced sacrificially and diverted to other areas during exercise. This process is less marked in the circumstance of exercise following ingestion of diet. As always, further studies of robust methodology would serve to improve the basis of this consensus.

The potential for longer-term adaptations in mesenteric flow in response to exercise training also presents an area requiring further exploration. Preoperative exercise has been shown to improve cardiorespiratory function [13, 62]. It is likely to confer change in other vascular beds, including the mesenteric arterial system. Preconditioning the mesenteric vasculature to the low flow states which may be encountered both during and after surgery for colonic resection through the use of exercise may reduce the risk of impaired anastomotic blood supply and subsequent healing. Studies examining mesenteric haemodynamics in response to regular exercise are therefore key to understanding the impact and potential utilisation of preoperative exercise in patients undergoing surgery which disrupts the normal mesenteric arterial supply.

## Appendix

See Tables 5 and 6.

**Table 5:** Pubmed literature review search conducted on 24.02.16

No.	Query	Expected results
#1	Hemodynamic* [tiab] OR Haemodynamic* [tiab] OR (blood [tiab] AND velocity* [tiab]) OR (blood* [tiab] AND flow* [tiab])	332,083
#2	Exercis* [tiab] OR (physical* [tiab] AND fitness [tiab]) OR (physical* [tiab] AND extert* [tiab]) OR (physical* [tiab] AND fit* [tiab])	227,879
#3	Doppler [tiab]	85,215
#4	(Splanchni* [tiab] OR mesenter* [tiab])	57,027
#5	(#1 AND #2 AND #3 AND #4)	33
#6	“Exercise”[Mesh] OR “Exercise Therapy”[Mesh] OR “Physical Exertion”[Mesh] OR “Physical Fitness”[Mesh]	206,385
#7	“Hemodynamics”[Mesh] OR “Blood Flow Velocity”[Mesh] OR “Regional Blood Flow”[Mesh]	610,249
#8	“Splanchnic Circulation”[Mesh] OR “Mesenteric Arteries”[Mesh]	28,105
#9	“Ultrasonography, Doppler”[Mesh]	57,969
#10	(#6 AND #7 AND #8 AND #9)	8
#11	8,036,905 [uid]	1
#12	13,356,576 [uid]	1
#13	3,596,339 [uid]	1
#14	9,824,727 [uid]	1
#15	Similar articles for PubMed (Select 8,036,905)	102
#16	Similar articles for PubMed (Select 13,356,576)	101
#17	Similar articles for PubMed (Select 3,596,339)	119
#18	Similar articles for PubMed (Select 9,824,727)	106
#19	(#15 OR #16 OR #17 OR #18)	343
#20	(#5 OR #10) NOT #19	23
#21	(#5 OR #10) NOT #19 Filters: English	23
#22	#19 Filters: Humans; English	204
#23	#22 OR #21	227

**Table 6:** Quality assessment using Down’s and Black Checklist

	Endo et al. [30]	Osada et al. [37]	Wright et al. [32]	Endo et al. [29]	Peters et al. [38]	Waaler et al. [41]	Osada et al. [43]	Perko et al. [39]	Puvi-Rajasingham et al. [33]	Puvi-Rajasingham et al. [44]
*Reporting*										
Is the hypothesis/aim/objective of the study clearly described?	1	1	1	1	1	1	1	1	1	1
Are the main outcomes to be measured clearly described in the introduction or methods section?	1	1	1	1	1	1	1	1	1	1
Are the characteristics of the patients included in the study clearly described?	0	1	1	0	1	1	1	1	1	0
Are the interventions of interest clearly described?	1	1	1	1	1	1	1	1	1	1
Are the distributions of principal confounders in each group of subjects to be compared clearly described?	0	0	1	0	0	0	1	0	0	0
Are the main findings of the study clearly described?	1	1	1	1	1	1	1	1	1	1
Does the study provide estimates of the random variability in the data for the main outcomes?	1	1	1	1	1	0	1	1	1	1
Have all important adverse events that may be a consequence of the intervention been reported?	0	0	1	0	0	0	0	0	0	0
Have the characteristics of patients lost to follow-up been described	1	1	1	1	1	1	1	1	1	1
Have actual probability values been reported for the main outcomes except where the probability value is less than 0.001?	0	0	1	0	0	0	1	0	0	0
*External validity*										
Were the subjects asked to participate in the study representative of the entire population from which they were recruited?	0	0	1	0	0	0	0	0	1	1
Were those subjects who were prepared to participate representative of the entire population from which they were recruited?	0	0	1	0	0	0	0	0	1	1
Were the staff, places, and facilities where the patients were treated, representative of the treatment the majority of patients receive?	0	0	0	0	0	0	0	0	1	1
*Internal validity bias*										
Was an attempt made to blind study subjects to the intervention they have received?	0	0	0	0	0	0	0	0	0	0
Was an attempt made to blind those measuring the main outcomes of the intervention?	0	0	0	0	0	0	0	0	0	0
If any of the results of the study were based on data dredging, was this made clear?	1	1	0	1	1	0	1	1	1	0
In trials and cohort studies, do the analyses adjust for different lengths of follow-up of patients, or in case–control studies, is the time period between the intervention and outcome the same for case controls?	0	0	1	0	0	1	0	0	0	0
Were the statistical tests used to assess the main outcomes appropriate?	1	1	1	1	1	1	1	1	1	1
Was compliance with the intervention/s reliable?	1	1	1	1	1	1	1	1	1	1
Were the main outcome measures used accurate (valid and reliable)?	1	1	1	1	1	1	1	1	1	1
*Internal validity*–*confounding (selection bias)*										
Were the patients in different intervention groups (trials and cohort studies) or were the cases and controls (case–control studies) recruited from the same population?	0	0	1	0	0	0	0	0	1	1
Were study subjects in different intervention groups (trials and cohort studies) or were the cases and controls (case–control studies) recruited over the same period of time?	0	0	1	0	0	0	0	0	0	0
Were the study subjects randomised to intervention groups?	0	0	0	0	0	0	0	0	0	0
Was the randomised intervention assignment concealed from both patients and health care staff until recruitment was complete and irrevocable?	0	0	0	0	0	0	0	0	0	0
Was there adequate adjustment for confounding in the analyses from which the main findings were drawn?	0	0	0	1	0	0	0	0	0	0
Were losses of patients to follow-up taken into account?	1	1	1	1	1	1	1	1	1	1
*Power*										
Did the study have sufficient power to detect a clinically important effect where the probability value for a difference being due to chance is less than 5%?	0	0	0	0	0	0	0	0	0	0
Total	11	12	19	12	12	11	12	12	16	14

	Puvi-Rajasingham et al. [34]	Duprez et al. [42]	Eriksen et al. [28]	Chauduri et al. [35]	Muller et al. [36]	Qamar et al. [40]
*Reporting*						
Is the hypothesis/aim/objective of the study clearly described?	1	1	1	1	1	1
Are the main outcomes to be measured clearly described in the introduction or methods section?	1	1	1	1	1	1
Are the characteristics of the patients included in the study clearly described?	1	1	1	0	1	1
Are the interventions of interest clearly described?	1	1	1	1	1	1
Are the distributions of principal confounders in each group of subjects to be compared clearly described?	0	0	0	0	0	0
Are the main findings of the study clearly described?	1	1	1	1	1	1
Does the study provide estimates of the random variability in the data for the main outcomes?	1	1	0	1	1	1
Have all important adverse events that may be a consequence of the intervention been reported?	0	0	0	0	0	0
Have the characteristics of patients lost to follow-up been described	1	1	1	1	1	1
Have actual probability values been reported for the main outcomes except where the probability value is less than 0.001?	0	1	0	0	1	1
*External validity*						
Were the subjects asked to participate in the study representative of the entire population from which they were recruited?	0	0	0	1	1	0
Were those subjects who were prepared to participate representative of the entire population from which they were recruited?	0	0	0	1	1	0
Were the staff, places, and facilities where the patients were treated, representative of the treatment the majority of patients receive?	0	0	0	1	1	0
*Internal validity bias*						
Was an attempt made to blind study subjects to the intervention they have received?	0	0	0	0	0	0
Was an attempt made to blind those measuring the main outcomes of the intervention?	0	0	0	0	0	0
If any of the results of the study were based on data dredging, was this made clear?	1	0	0	1	0	0
In trials and cohort studies, do the analyses adjust for different lengths of follow-up of patients, or in case–control studies, is the time period between the intervention and outcome the same for case controls?	0	0	0	0	0	0
Were the statistical tests used to assess the main outcomes appropriate?	1	1	1	1	1	1
Was compliance with the intervention/s reliable?	1	1	1	1	1	1
Were the main outcome measures used accurate (valid and reliable)?	1	1	1	1	1	1
*Internal validity*–*confounding (selection bias)*						
Were the patients in different intervention groups (trials and cohort studies) or were the cases and controls (case–control studies) recruited from the same population?	0	0	0	1	1	0
Were study subjects in different intervention groups (trials and cohort studies) or were the cases and controls (case–control studies) recruited over the same period of time?	0	0	0	0	0	0
Were the study subjects randomised to intervention groups?	0	0	0	0	0	0
Was the randomised intervention assignment concealed from both patients and health care staff until recruitment was complete and irrevocable?	0	0	0	0	0	0
Was there adequate adjustment for confounding in the analyses from which the main findings were drawn?	0	0	0	0	0	0
Were losses of patients to follow-up taken into account?	1	1	1	1	1	1
*Power*						
Did the study have sufficient power to detect a clinically important effect where the probability value for a difference being due to chance is less than 5%?	0	0	0	0	0	0
Total	12	12	10	15	16	12
